# A Comparison of Discrete Crack and Smeared Crack Methods Applied to CFRP/Al Riveting Damage Modeling

**DOI:** 10.3390/ma18194511

**Published:** 2025-09-28

**Authors:** Minghao Zhang, Kun Tian, Zengqiang Cao, Tong-Earn Tay

**Affiliations:** 1 School of Mechanical Engineering, Northwestern Polytechnical University, Xi’an 710072, China; zhang_minghao@mail.nwpu.edu.cn (M.Z.); czq66326@nwpu.edu.cn (Z.C.); 2 School of Aerospace Engineering and Applied Mechanics, Tongji University, Shanghai 200092, China; 3 Department of Mechanical Engineering, National University of Singapore, 9 Engineering Drive 1, Singapore 117565, Singapore

**Keywords:** CFRP, riveting damage, discrete crack method, floating node method, smeared crack method

## Abstract

Carbon-fiber-reinforced-polymer/aluminum (CFRP/Al) double-sided countersunk riveted joint is a key joining technology for lightweight and high-performance aircraft structures. Advanced numerical simulation techniques are helpful in predicting riveting damage evolution and the optimization of the joining process. In this study, a discrete crack modeling (DCM) method based on the floating node method (FNM) was employed to investigate the initial riveting damage behavior and interference characteristics during the electromagnetic riveting (EMR) process with five cases of rivet-hole clearances. The results were compared with those obtained from the conventional smeared crack method (SCM). The findings show that the interference distribution along the axial direction of the joint is non-uniform, and increasing the rivet-hole clearance helps alleviate the initial riveting damage. The FNM accurately modeled the initiation and propagation of matrix cracks and delamination, albeit at the cost of some computational efficiency.

## 1. Introduction

Carbon fiber reinforced polymer (CFRP)/metal stack-up structures play a crucial role in modern aircraft design and manufacturing. Lightweight thin-walled structures with integrated functions and cost-effective fabrication are often achieved by combining aluminum alloy (Al) frames, which have excellent machinability, with composite panels [1,2]. The CFRP/Al double-sided countersunk riveting technique is a key joining method for such heterogeneous material structures [3,4]. This type of joint ensures surface flushness, which helps reduce airflow separation and drag, thereby improving fuel efficiency. At the same time, it meets both weight reduction and airtightness requirements, and thus is widely used in fuselage sections, wing surfaces, cabin doors, and other parts [5].

**Figure 1:**
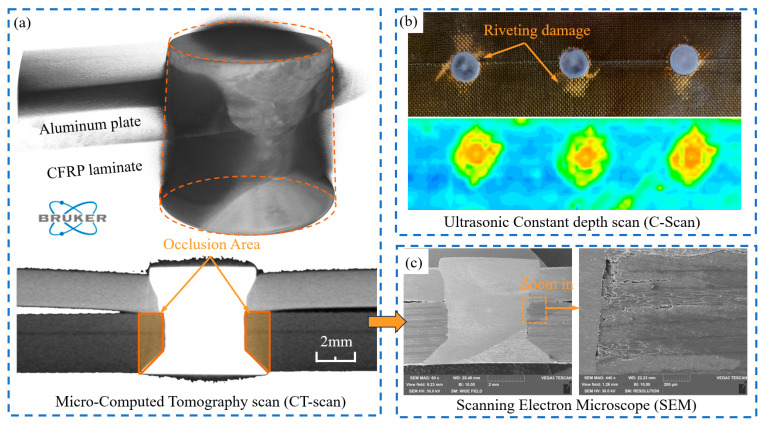
Damage detection method for CFRP/Al countersunk riveted joint: (**a**) Micro-CT; (**b**) C-Scan; (**c**) SEM.

The quality of riveting and joint performance largely depends on the manufacturing process [6], especially for CFRP/Al countersunk riveting joints, which are complex. Compared to straight holes, countersunk holes exhibit higher stress concentrations [7]. Moreover, since CFRP exhibits little ductile behavior, improper process parameters may cause the rivet to undergo plastic deformation, leading to severe initial damage to the CFRP [3,8]. Such damage can further propagate under external loads, eventually resulting in premature structural failure [9]. Therefore, clarifying the damage mechanisms in CFRP during riveting and controlling the initiation of damage are critical for ensuring the safety and reliability of aircraft structures. However, in CFRP/Al riveted joints, nondestructive detection of initial assembly damage in practice remains highly challenging. As shown in Figure 1, for Micro-Computed Tomography (CT) techniques, the attenuation of X-rays varies significantly due to differences in material densities, often resulting in signal oversaturation in the metallic region but obscuring the damage signals from the CFRP [10]. For C-scan ultrasonic techniques, acoustic impedance mismatch and interface reverberation effects can overlap with actual damage signals, resulting in poor detection accuracy [11]. Furthermore, the conical part of the countersunk rivet creates a cylindrical obstruction zone, making it even more difficult to detect critical damage areas. Although destructive methods such as scanning electron microscopy (SEM) can clearly reveal initial damage, the process of observing the cross-section may introduce additional damage [12]. Therefore, employing numerical simulation has become a complementary approach to assess initial assembly damage in composite materials.

In aircraft manufacturing, the geometric changes in fastening joints after assembly are often among the most critical process parameters. These parameters are typically defined by interference size, expansion size, or fastener-hole clearance [8]. Parametrized experimental studies are often expensive and difficult. The Smeared Crack Method (SCM), based on Continuum Damage Mechanics (CDM), is widely used in finite element analysis (FEA) of initial assembly damage in composite materials at the mesoscale or macroscale [13]. The core concept of this method is to homogenize the mechanical effects of discrete cracks—primarily stiffness degradation—across entire elements or integration points. Researchers have compared the damage distribution of CFRP bolted joints predicted by SCM and found that using thin layers [14] and adding bushings [15] can reduce the initial CFRP damage and increase the allowable interference size. Li et al. [16] developed a CFRP/Al blind rivet joints model and concluded that although the insertion process of interference fit can cause damage around the hole, it enhances the bearing strength of the joint. SCM has also been widely applied in numerical simulations of CFRP/Al self-piercing riveting (SPR) [17]. However, when multiple failure modes occur in a coupled manner, SCM cannot distinguish and quantify the contribution of each mode solely through the stiffness degradation of the elements. Additionally, the accuracy of SCM results is highly dependent on mesh size and orientation [18]. When more precise interrogation of local failure details and crack propagation behavior is required, the discrete crack method (DCM) is a better choice. Existing DCMs include methods such as the Extended Finite Element Method (XFEM) and the Phantom Node Method (PNM), but these methods are limited by the complexity of geometric mapping and integration [19]. Funari et al. [20,21] employed an adaptive/remeshing strategy that introduces a highly refined discretization ahead of the crack tip to simulate discontinuous behavior. Although this approach is better suited for dynamic propagation dominated by a single crack, such as debonding, it is not applicable to other scenarios. Chen et al. [19] proposed the Floating Node Method (FNM), which enables better handling of complex multiple cracks modeling problems through topological reconfiguration of floating degrees of freedom. Lu et al. [22] further refined the technique by developing the Adaptive Floating Node Method (A-FNM). Zhi et al. [23] significantly improved the predictive capability for complex failure mechanisms under compressive/impact loads by incorporating geometric nonlinearity and inclined crack treatment into the modeling approach. Tian et al. [24,25] addressed the convergence issues associated with implicit FE by employing the FNM algorithm for explicit FE. In aerospace applications involving multi-component and multi-material assemblies, the insertion of cracks coupled with strong multi-interface contact interactions may trigger large-scale out-of-plane instabilities and convergence difficulties, necessitating specific treatments for contact behavior.

In this study, the effects of different rivet-hole clearances on the interference characteristics and damage behavior of CFRP/Al double-sided countersunk riveted joints during the manufacturing process were investigated by considering five clearance conditions. Numerical simulations were conducted using both the SCM and DCM within the explicit finite element (FE) framework. The influence of clearance on interference distribution and CFRP damage behavior was analyzed and compared with results from electromagnetic riveting (EMR) experiments for validation. Special attention was given to the differences between SCM and DCM in terms of rivet forming simulation, damage mode prediction, and computational efficiency. This study aims to provide a reference for selecting appropriate numerical modeling methods in aircraft design.

## 2. Numerical Simulation Method

### 2.1. FNM Theory

**Figure 2:**
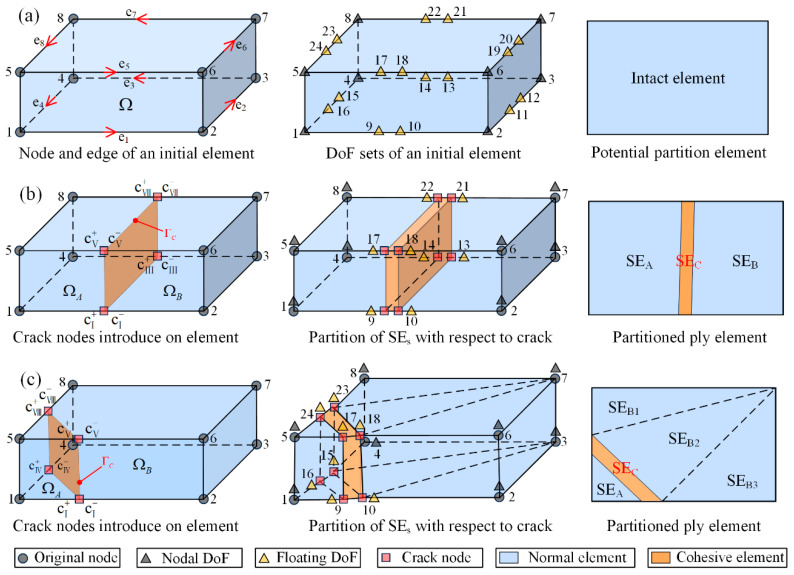
FNM principle [19,26]: (**a**) definition of potential partition element, (**b**) opposite-edge crack propagation mode, (**c**) adjacent-edge crack propagation mode.

The DCM modeling method in this study is implemented based on the FNM. In standard FEA, the brick elements solution process is governed by the continuum assumption, where the degrees of freedom (DoFs) are inherently nodal, and the solution over the rest of the domain is interpolated from these nodal values using shape functions. When there is the initiation and propagation of cracks, the original domain becomes discontinuous along the crack boundary, requiring additional DoFs and interpolation functions to represent the motion of the new crack boundary. To address this, the FNM partitions element definition by incorporating not only nodal connectivity but also edge/surface connectivity by introducing floating nodes [24]. The additional DoFs of these floating nodes represent the displacements of crack nodes on geometrical entities, although the DoFs must not coincide with initial nodes. As illustrated in Figure 2a, the floating nodes of the potential partition element typically stay at the origin, are not assigned coordinates, and do not participate in FE calculations. Only when the element’s stress or strain satisfies the failure criterion are the floating nodes’ coordinates and DoFs activated and allocated to specific elements. This flexibility enables precise capture of crack propagation processes.

Once a potential partition element satisfies the failure criterion, the floating DoFs on its edges are activated to form a cohesive crack element, partitioning the initial element into multiple sub-elements. It is assumed that only one crack can traverse each partitioned element, and cracks cannot pass through original nodes. Consequently, two crack propagation modes exist within a partitioned element: opposite-edge propagation, as illustrated in Figure 2b, and adjacent-edge propagation, as shown in Figure 2c. After partitioning, the element stiffness and force matrices are reassembled using the DoFs from the sub-elements and cohesive element. Detailed mathematical derivations can be found in the literature [26]. The crack propagation mechanism follows the edge status variable approach [26], whereby once a propagation criterion is met at a specific edge, its status is updated from crack tip to crack wake. Neighboring elements detect this updated status through their shared edges, enabling automatic crack advancement across the mesh.

**Figure 3:**
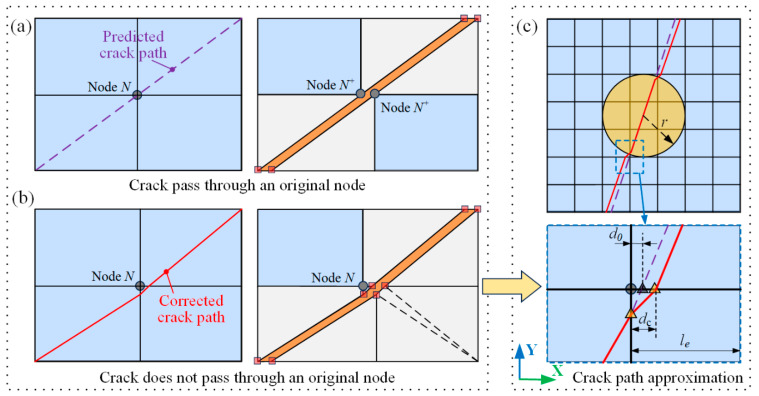
Crack propagation path correction [25]: (**a**) crack pass through an original node; (**b**) corrected path; (**c**) offset definition.

During the crack propagation process, the crack path is not allowed to penetrate original nodes, as illustrated in Figure 3a, Node N splits into two Nodes N^+^, a behavior that cannot be captured solely with floating nodes in codes. Therefore, any predicted crack path that may cross an original node must be corrected, as illustrated in Figure 3b. A path offset of 10% of the element length *l*_e_ is applied to any theoretically predicted crack path intersecting an original node [25], as depicted in Figure 3c. Furthermore, a minimum spacing between adjacent cracks is enforced, set to be at least 1.5 times the size of the element.

### 2.2. Progressive Damage Modeling

To accurately predict the damage behavior in the CFRP laminate during the riveting process, a combination of failure criteria was adopted within a progressive damage modeling framework to account for fiber damage, matrix damage, and delamination separately. It is important to note that to highlight the differences in modeling methods, both the SCM and FNM employed the same damage onset criteria. The delamination behavior is modeled using a Cohesive Zone Model (CZM), with damage onset described by the bilinear traction–separation law. However, the key distinction lies in the description of the damage evolution behavior.

For the SCM, the evolution of fiber and matrix damage is governed by energy-based linear softening laws. For the FNM introduced in Section 2.1, due to the algorithmic constraint that only one crack can be inserted per partitioned element, the description of localized and abrupt fiber cracks still adopts the same linear softening law as used in SCM. However, both matrix cracks and delamination are captured through the insertion of cohesive elements, with their damage evolution governed by the linear softening law, and the mixed-mode fracture energy is determined by a power-law criterion of the CZM. The FNM primarily focuses on densely distributed, morphologically complex matrix cracks exhibiting significant local effects. The specific damage models adopted in this study are introduced in this section.

#### 2.2.1. Onset of Damage Model

(1) Onset of fiber damage model

The damage dominated by fiber under tensile or compressive stress is described using the maximum stress criterion [27] along the fiber direction:

(1) $$\frac{\sigma_{11}}{X_{T}}=1,\;\frac{\sigma_{11}}{X_{C}}=-1$$

where *σ*_11_, *X*_T,_ and *X*_C_ are the fiber direction stress, fiber direction tensile strength, and fiber direction compressive strength, respectively.

(2) Onset of matrix damage model

It is assumed that matrix cracking initiates when the following quadratic interaction criterion is met as follows [28]:

(2) $$\left\{\begin{array}{cc} {\left(\frac{\sigma_{N}}{Y_{T}}\right)}^{2}+{\left(\frac{\tau_{N}}{S_{T}}\right)}^{2}+{\left(\frac{\tau_{L}}{S_{L}}\right)}^{2}=1 & \sigma_{n}\geq 0\\ {\left(\frac{\tau_{T}}{S_{T}-\mu_{T}\sigma_{n}}\right)}^{2}+{\left(\frac{\tau_{L}}{S_{L}-\mu_{L}\sigma_{n}}\right)}^{2}=1 & \sigma_{n}<0 \end{array}\right.$$

where *Y*_T_ is transverse tensile strength, *S*_T_ and *S*_L_ are transverse and longitudinal shear strength, respectively. *μ*_T_ and *μ*_L_ are friction-like parameters. The traction components at the crack surface based on the Mohr–Coulomb criterion are given by [29]:

(3) $$\left\{\begin{array}{l} \sigma_{N}=\frac{\sigma_{22}+\sigma_{33}}{2}+\frac{\sigma_{22}-\sigma_{33}}{2}\cos\left(2\alpha \right)+\tau_{23}\sin\left(2\alpha \right)\\ \tau_{T}=-\frac{\sigma_{22}-\sigma_{33}}{2}\sin\left(2\alpha \right)+\tau_{23}\cos\left(2\alpha \right)\\ \tau_{L}=\tau_{12}\cos\left(\alpha \right)+\tau_{31}\sin\left(\alpha \right) \end{array}\right.$$

where *σ*_22_, *σ*_33_, *τ*_12_, *τ*_23_, and *τ*_31_ are the stress components with local material coordinates, and *α* is the fracture plane angle.

(3) Onset of delamination model

The CFRP laminate interface between two plies was simulated by inserting Cohesive elements. The delamination initiation criterion is written in a quadratic form [13]:

(4) $$\left\{\begin{array}{cc} {\left(\frac{\tau_{3}}{N}\right)}^{2}+{\left(\frac{\tau_{1}}{S}\right)}^{2}+{\left(\frac{\tau_{2}}{T}\right)}^{2}=1 & \tau_{3}\geq 0\\ {\left(\frac{\tau_{1}}{S}\right)}^{2}+{\left(\frac{\tau_{2}}{T}\right)}^{2}=1 & \tau_{3}<0 \end{array}\right.$$

where *τ*_3_ refers to the interlaminar normal stress for mode I delamination, *τ*_1_ and *τ*_2_ correspond to the shear stress required for mode II and III delamination, respectively, *N*, *S,* and *T* refer to the corresponding interlaminar interface strengths.

#### 2.2.2. Damage Evolution

(1) Fiber damage evolution

Assuming the energy is dissipated by the fiber crack smeared over the volume of the element [23], the energy balance of this process can be defined as follows:

(5) $$\int_{0}^{\varepsilon_{fc}^{f}}\sigma_{11}d\varepsilon_{fc}=\frac{G_{fc}}{le}$$

where $\varepsilon_{fc}$ is the fiber longitudinal strain, and the final failure longitudinal strain $\varepsilon_{fc}^{f}$ is related to the fiber longitudinal fracture toughness *G_fc_*:

(6) $$\varepsilon_{fc}^{f}=\frac{2G_{fc}}{X_{C}l_{e}}$$

The fiber damage evolution follows a linear law of degradation, and the fiber damage index *d_f_* is defined as follows [30]:

(7) $$d_{f}=\frac{\varepsilon_{fc}^{f}(\varepsilon_{fc}-\varepsilon_{fc}^{0})}{\varepsilon_{fc}(\varepsilon_{fc}^{f}-\varepsilon_{fc}^{0})},\;0<d_{f}<1$$

where $\varepsilon_{fc}^{0}$ is the initial fiber strain, and the damage index is used to characterize the linear softening process in damage modeling, with 0 representing no damage and 1 representing complete failure.

(2) Matrix damage evolution for SCM

To facilitate the hybrid failure softening calculations, the characterization is performed by defining the following effective stress *σ_eff_* and displacements *d_eff_* [31]:

(8) $$\sigma_{eff}=\sqrt{{\langle \sigma_{22}\rangle }^{2}+\tau_{12}^{2}}$$

(9) $$d_{eff}=\frac{2\left(G_{n}+G_{s}\right)}{\sigma_{eff}}$$

(10) $$G_{n}=\frac{1}{2}\langle \sigma_{22}\rangle \langle \varepsilon_{22}\rangle l_{e},\;G_{s}=\frac{1}{2}\tau_{12}\gamma_{12}l_{e}$$

The linear softening law is then defined by the effective stress and displacement based on the energy criterion:

(11) $$\int_{0}^{d_{eff}}\sigma_{eff}d\left(d_{eff}\right)=G_{mc}$$

where the mixed-mode fracture energy G*_mc_* was obtained from the Benzeggagh–Kenane (B-K) criterion [32]:

(12) $$G_{mc}=G_{nc}+\left(G_{sc}-G_{nc}\right)B^{\eta }$$

(13) $$B=\frac{G_{s}}{\left(G_{n}+G_{s}\right)}$$

where *G_mc_* is matrix mixed mode fracture energy, *G_nc_* and *G_sc_* represent the mode-Ⅰ and mode-II fracture toughness, respectively, and *η* is the semi-empirical material fracture criterion exponent.

From the fracture toughness, the final matrix failure strain $\varepsilon_{m}^{f}$ can be calculated as follows:

(14) $$\varepsilon_{m}^{f}=\frac{2G_{mc}}{\sigma_{eff}l_{e}}$$

Similarly to the degradation law used in fiber damage evolution, the matrix damage index *d_m_* is defined as follows:

(15) $$d_{m}=\frac{\varepsilon_{m}^{f}(\varepsilon_{m}-\varepsilon_{m}^{0})}{\varepsilon_{m}(\varepsilon_{m}^{f}-\varepsilon_{m}^{0})},\;0<d_{m}<1$$

where $\varepsilon_{m}$ and $\varepsilon_{m}^{0}$ represent the matrix strain and initial matrix strain, respectively. Given the coupled behavior between fiber and matrix damage, the combined degradation scheme is used to denote in-plane damage failure [31]:

(16) $$\left\{\begin{array}{l} E_{11}=\left(1-d_{f}\right)E_{11}^{0}\\ E_{22}=\left(1-d_{f}\right)\left(1-d_{m}\right)E_{22}^{0}\\ G_{12}=\left(1-d_{f}\right)\left(1-d_{m}\right)G_{12}^{0}\\ \nu_{12}=\left(1-d_{f}\right)\left(1-d_{m}\right)\nu_{12}^{0} \end{array}\right.$$

where 0 represents the initial material properties before degradation, *d_f_* and *d_m_* are the damage indices in Equations (7) and (15), respectively.

(3) CZM damage evolution

For damage evolution following delamination in the SCM, as well as for matrix cracking and delamination in the FNM, the mixed-mode fracture energy is modeled by a power-law criterion [23]:

(17) $${\left(\frac{G_{n}}{G_{nc}}\right)}^{\eta }+{\left(\frac{G_{s}}{G_{sc}}\right)}^{\eta }+{\left(\frac{G_{t}}{G_{tc}}\right)}^{\eta }=1$$

where *G_n_*, *G_s_*, and *G_t_*, are the current fracture energies in the normal and shear directions, *G_nc_*, *G_sc_*, and *G*_tc_ are the corresponding critical fracture energies.

### 2.3. EMR Experiment

**Figure 4:**
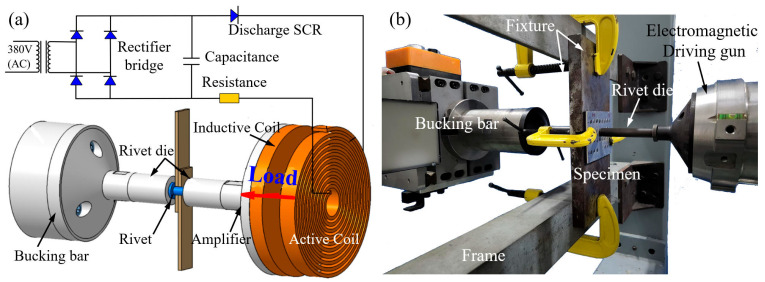
EMR technology: (**a**) physical principle of the EMR, (**b**) experimental setup for EMR [9].

The CFRP/Al double-sided countersunk riveting experiments in this study employed EMR technology [8,9]. The working principle is illustrated in Figure 4a. A pulse current from the discharge of the capacitor generates a force propelling the gun to deliver a high-velocity impact to the rivet in contact with the bucking bar. The rivet undergoes high strain-rate plastic deformation and forms an interference-fit joint. This process typically lasts from several hundred microseconds to a few milliseconds [33].

To investigate the influence of rivet-hole clearance on the initial damage in the CFRP during the manufacturing process, five values of clearance were defined in Table 1. Standard HB6478 4 × 8 rivets were used, and different clearance values were achieved by varying the hole diameter in the CFRP/Al stack-up structure. The riveting experiments were conducted using the EMR-1000 EMR system developed by Northwestern Polytechnical University, as shown in Figure 4b. A stack-up structure consisting of a 3.3 mm CFRP laminate and a 2 mm Al plate was fixed in a clamping fixture. Under the action of the electromagnetic driving gun, the rivet formed a driven head on the Al side, completing the EMR process.

The post-riveting diameter of the rivet is difficult to measure directly. Therefore, the interference size is often indirectly controlled using parameters such as rivet shank length [34], driven head dimensions [35] or rivet-hole clearance [8]. To obtain a relatively accurate measurement of the interference size and to observe the initial damage distribution, it is necessary to dissect the joint after the EMR process has been completed. And the interference size *I* is defined as follows [36]:

(18) $$I=\frac{d_{n}-D}{D}\times 100\%$$

where *d*_n_ is the diameter of the expanded rivet and *D* is the rivet-hole diameter.

During the interference measurement, the edge region of the riveted joint is first carefully cut using a saw. Then, the joint is ground and polished using a metallographic grinder to expose the central cross-section at half the diameter, where the expanded rivet diameter *d*_n_ is measured. Since the rivet expands unevenly along the axial direction, to separately obtain the interference sizes in the Al plate and the CFRP laminate, measurements are taken at depths of 0.8 mm and 1.4 mm in the Al plate, and at depths of 1.45 mm and 2.9 mm in the laminate, following the standard GJB715.25-90 [37] and using an optical microscope. After measurement, the two values from each side (Al or CFRP) were averaged. For each rivet-hole clearance group, three specimens were measured to minimize experimental error. The interference sizes for the Al or laminate side were then calculated as the average of the six measurements (3 specimens × 2 positions). The measurement data results are shown in the Appendix A. Finally, the CFRP damage distribution was observed using SEM for this cross-section.

### 2.4. FE Analysis

#### 2.4.1. FE Model

**Figure 5:**
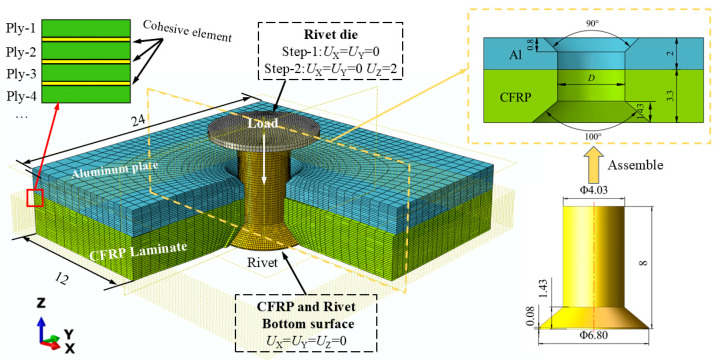
FE model for the CFRP/Al double-sided countersunk electromagnetic riveting simulation [Unit: mm].

An Abaqus/Explicit FE model for the CFRP/Al riveted joint is shown in Figure 5, where a quarter of the FE model is hidden to show the internal initial assembly configuration. Under the axial compressive load of the rivet die, the driven head was formed on the Al plate side. The bottom surface of the CFRP laminate and the surface of the rivet manufactured head were with displacement constraints. After the rivet formation was completed, the die retracted, allowing the material to undergo localized elastic recovery. The contact behavior between the four parts is modeled using surface-to-surface master-slave contact pairs, with the contact pairs and friction coefficients shown in Table 2. The region around the rivet hole was identified as a critical damage zone. Based on previous FE studies [38] and preliminary tests with different mesh sizes, a mesh size of 0.2 mm was ultimately selected as the finest mesh achievable within the current computational resources. Identical FE meshes were used for both SCM and FNM modeling. The elements used in the SCM laminate model are of type C3D8R (eight-node linear brick element, reduced integration), while the partitioned elements in the FNM model are user-defined elements implemented via Abaqus VUEL (User Element for Explicit) subroutine. These user-defined elements have 8 conventional nodes, with the floating nodes only activated and assigned to the element when cracking occurs. For interlaminar interface modeling, both the FNM and SCM models implemented cohesive elements pre-inserted between the ply elements. The composite failure criteria for both SCM and FNM modeling are described in Section 2.2 and invoked via a VUMAT (Vectorized User-defined Material) subroutine. The boundary conditions, material properties, and element types for the metal part are identical in both models. The type of countersunk rivets used in this study is HB6478-4 × 8, and the geometry of the rivet-hole is shown in Figure 5. The simulated rivet-hole clearance variables are defined in the same way as those listed in Table 1. Different clearance values are obtained by assembling rivets of identical diameter into models with varying hole diameters in the Al plate and CFRP laminate.

#### 2.4.2. Material Properties and Constitutive Equation for Metal

The composite material used in this study is carbon fiber reinforced bismaleimide resin matrix composite ZT7H/QY9611, with a layup sequence of [90/−45/0/45/45/0/−45/90/45/0/−45/90/−45/0/45/90/−45/0/45/45/0/−45/90/45] consisting of 24 plies. The nominal ply thickness is 0.125 mm. The material properties of the ZT7H/QY9611 composite laminate and the CZM parameters are detailed in Table 3 and Table 4, respectively. These parameters are equivalent in both models.

In conventional pressure riveting, the deformation of the rivet material is primarily characterized by homogeneous slip, whereas in the EMR process, the rivet undergoes rapid plastic deformation within an extremely short duration, exhibiting characteristics of adiabatic shear deformation [44]. Therefore, it is necessary to consider the strain rate effect and thermal softening in the FEA of the EMR process [45]. To characterize the plastic deformation behavior of metallic materials under these conditions, the Johnson–Cook (J-C) constitutive equation was adopted. The pure titanium TA1 rivet J-C model is as follows [46]:

(19) $$\sigma =(260+488\varepsilon^{0.5})(1+0.1\ln{\dot{\varepsilon }}^{*})(1-{\left(\frac{T-T_{r}}{T_{m}-T_{r}}\right)}^{1.4})$$

Since the deformation of the Al plate is significantly smaller than that of the rivet, and its heat dissipation capacity is much greater than that of the forming zone of the driven head, the 2A12 J-C model is provided without considering thermal softening effects [47]:

(20) $$\sigma =(380+580\varepsilon^{0.46})(1+0.03\ln{\dot{\varepsilon }}^{*})$$

where *σ* is the Von Mises flow stress, *ε* is the equivalent plastic strain, *T*_r_ is the reference temperature (25 °C), and *T*_m_ is the melting point, which is 620 °C for 2A12 and 1668 °C for TA1. Strain rate ratio ${\dot{\varepsilon }}^{*}$ is written as follows:

(21) $${\dot{\varepsilon }}^{*}=\dot{\varepsilon }/{\dot{\varepsilon }}_{0}$$

where $\dot{\varepsilon }$ is the actual strain rate, ${\dot{\varepsilon }}_{0}$ is the static tensile strain rate.

Other mechanical properties are listed in Table 5. Since the stiffness of the rivet die is significantly greater than that of the rivet, it is assumed to be a rigid body without deformation during the simulation process.

## 3. Results and Discussion

### 3.1. Riveting Deformation Process

**Figure 6:**
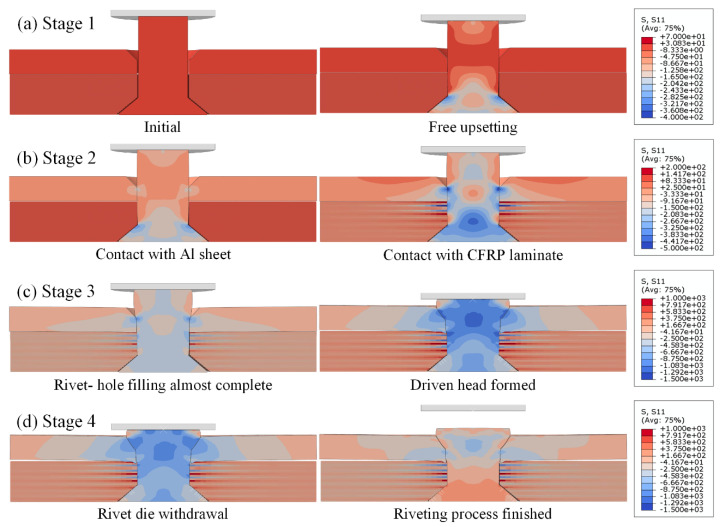
EMR process with FNM modeling result: (**a**) stage 1; (**b**) stage 2; (**c**) stage 3; (**d**) stage 4.

**Figure 7:**
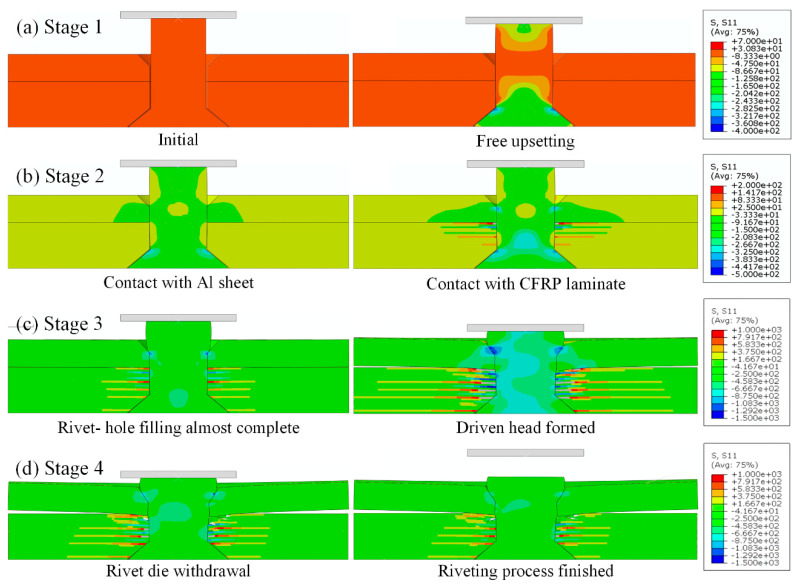
EMR process with SCM modeling result: (**a**) stage 1; (**b**) stage 2; (**c**) stage 3; (**d**) stage 4.

The study of initial riveting damage in CFRP cannot be separated from the analysis of material flow and deformation behavior of the rivet during the riveting process. Figure 6 and Figure 7 illustrate the EMR process of CFRP/Al joints using the FNM and SCM modeling approaches, respectively. It should be noted that, since ABAQUS visualization cannot process the output results of user-defined elements, the FNM simulation results are displayed using ParaView 5.13. The modeling procedures for the metal parts are identical in both SCM and FNM. Therefore, to focus on comparing the stress variations in the CFRP laminates during the riveting process, the normal stress in the fiber direction (S11, fiber-direction stress) was selected for comparison, with the same stress scale applied in both cases.

According to the material flow trend of the rivet, the EMR process is simplified into four stages. In stage 1, the rivet undergoes elastic deformation followed by plastic deformation under the action of the riveting force. The shank exhibits a typical barrel-shaped profile characteristic of free upsetting. This stage involves only rivet material deformation. In stage 2, the radial extrusion stage, the middle part of the rivet shank first comes into contact with the transition area between the countersunk hole and the straight hole in the Al plate. The hole wall experiences elastic deformation followed by plastic deformation. Contact with the laminate hole wall occurs only after full engagement with the Al hole wall. Material flow remains predominantly axial during this phase. In stage 3, the driven head forming stage, the rivet material mainly flows into the countersunk hole. Friction between the driven head and the Al countersink redirects the material flow radially. Meanwhile, the contact pressure at the driven head-hole interface in the Al plate is transferred downward into the laminate. This has two effects. On the one hand, this causes Al plate warpage deformation and forms a rivet gap. Additionally, relative sliding between the rivet and the Al plate creates more space for the rivet to expand at the aluminum/laminate interface. On the other hand, severe compressive deformation occurs at the top laminate plies. Once the countersunk hole is nearly filled, some of the material is forced into the straight hole part of the laminate, leading to secondary upsetting and exacerbating damage in the CFRP. In the stage 4 rebound stage, after rivet formation, the die is retracted, and the assembly undergoes elastic rebound, generating residual stresses in the joint [48], completing the EMR process.

**Figure 8:**
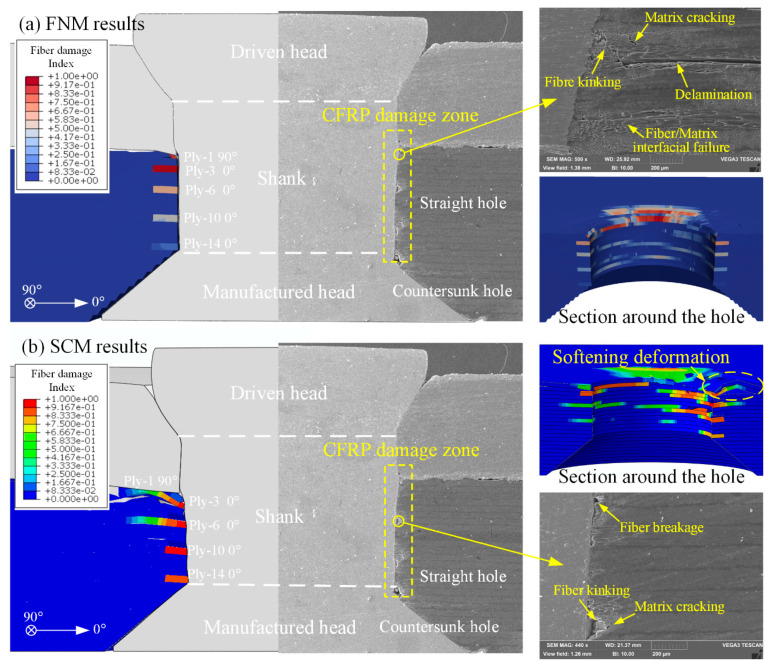
Comparison between fiber damage contour and SEM cross-section of joint damage: (**a**) FNM results; (**b**) SCM results.

By comparing Figure 6 and Figure 7, it can be observed that the S11 stress distribution trends and scale ranges in the FNM and SCM models during the EMR process are generally similar. Therefore, a further comparison was conducted using the fiber damage contour plots, which are directly related to S11, along with SEM observation results, as shown in Figure 8.

From the SEM results, it can be observed that CFRP riveting damage primarily occurs along the hole wall in the straight hole part, exhibiting fiber kinking and breakage caused by the rivet extrusion, accompanied by local matrix cracking and delamination. In contrast, the rivet-manufactured head within the countersunk hole of the laminate undergoes minimal plastic deformation during the rivet expansion process. As a result, the countersunk hole part of the CFRP laminate remains basically intact, with almost no visible damage.

The fiber damage contour plots from both the FNM and SCM show good agreement with the SEM results. On the joint’s cross-section in the 0° direction, fiber damage was observed not only in the first 90° ply but also in the 0° plies of the 3rd, 6th, 10th, and 14th plies. The predictions from both modeling methods are generally consistent. However, a further comparison of the fiber damage contours around the hole area of the laminate shows that, due to stiffness degradation during the progressive damage process in the SCM model, element distortion occurs around the hole. In contrast, the hole wall in the FNM model shows no significant distortion of elements.

### 3.2. Interference Behavior

**Figure 9:**
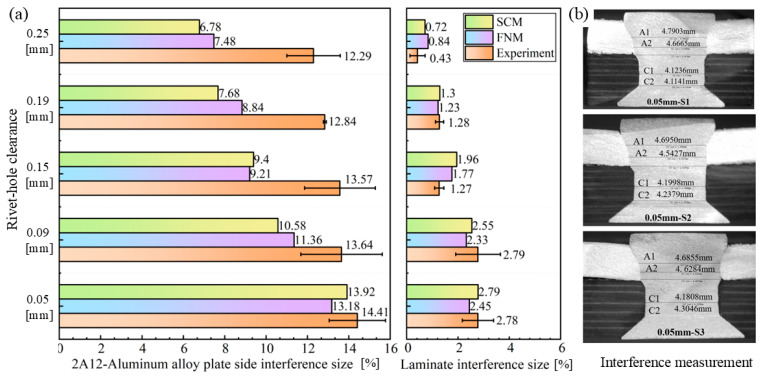
Interference sizes of riveted joints: (**a**) comparison of interference sizes obtained from SCM, FNM, and experiments, (**b**) interference measurement method (example with 0.05 mm clearance).

The relative interference size is a critical parameter in aircraft assembly manufacturing. Interference-fit joints rely on the normal contact force generated by elastic deformation between mating surfaces to transfer loads, where the contact pressure is directly determined by the relative interference size [49]. Moderate interference size can introduce residual compressive stress to inhibit the initiation and propagation of fatigue cracks, and excessive interference size may exacerbate stress concentration and initial material damage [50,51]. Figure 9a shows the interference size obtained by the SCM and FNM and compares them with experimental results. Figure 9b shows an example of the interference size measurement for the Al plate and the CFRP laminate.

The standard deviation error bars in Figure 9a indicate the variability across three repeated measurements, showing overall errors and fluctuations in the measurements of the interference size. These are caused by a combination of factors, including drilling inaccuracies, potential inconsistencies in repeated riveting experiments, imprecise grinding during sectioning that may not reach the maximum diameter of the joint, and measurement errors. Accurate measurement of the interference size and CFRP damage assessment after riveting face similar challenges, but repeated experiments can clearly help to minimize these errors.

From the experimental and simulated measurements, the interference size on the Al plate side is significantly higher than that on the CFRP laminate side. This reflects that the interference or deformation behavior along the axial direction of the rivet is non-uniform, with material flowing into the rivet hole gradually decreasing from top to bottom, and increasing the initial rivet hole clearance can effectively reduce the joint’s interference size. The difference in interference results between SCM and FNM simulations is minimal, as the primary distinction between these methods pertains only to composite material modeling. Compared with the experimental results, both SCM and FNM deliver engineering-acceptable predictions for interference size on the CFRP laminate side. It is generally recognized that the interference size in composite joints should be kept below 2% of the nominal diameter increase after riveting [52]. According to both simulation and measurement results, a rivet-hole clearance greater than 0.15 mm meets the process requirement of keeping the laminate side interference size below 2%. However, the FE model failed to accurately predict the interference behavior of the Al plate. In addition to the experimental errors mentioned above, the discrepancies between the simulation and experimental results may also be influenced by a combination of factors, such as mesh size, the simplified constitutive model of the metallic material, or complex interfacial friction behavior. Identifying the potential causes would require a comprehensive sensitivity analysis, which in turn demands substantial computational resources. These issues represent a limitation of the cases studied in this paper.

### 3.3. CFRP Damage Analysis

**Figure 10:**
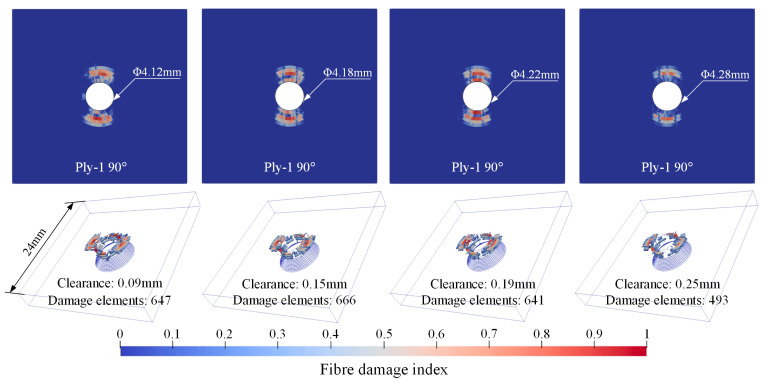
Fiber damage distribution contour from FNM.

**Figure 11:**
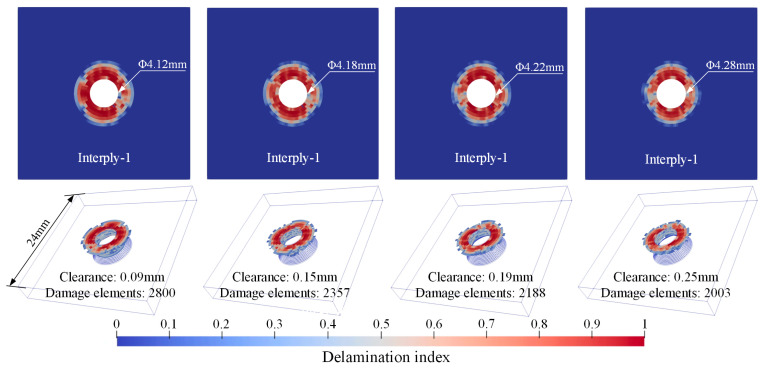
Delamination distribution from FNM.

**Figure 12:**
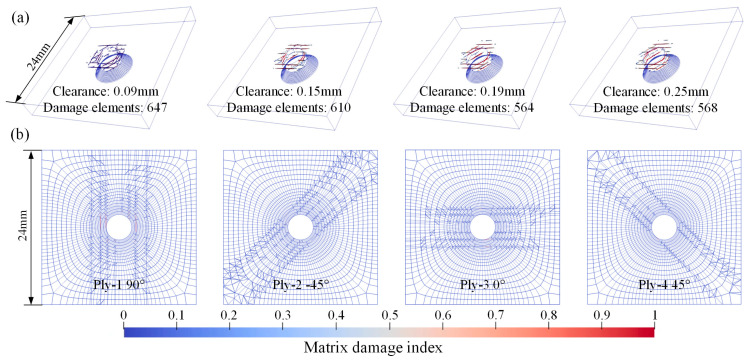
Matrix damage distribution contour from FNM: (**a**) matrix damage contour with different clearances; (**b**) matrix crack propagation prediction paths for the top four plies.

Figure 10, Figure 11 and Figure 12, respectively, present the initial riveting-induced fiber damage, delamination, and matrix damage results obtained from FNM simulations under different rivet-hole clearances. It can be seen that all damage is concentrated in the straight hole part, with no damage occurring in the countersunk hole part. Regardless of the rivet-hole clearance, fiber and delamination damage decrease progressively through the thickness direction from top to bottom. The most severe damage occurs in the topmost 90° ply. Cohesive elements representing matrix cracks are relatively uniformly distributed around the hole, as illustrated in Figure 12a. During rivet expansion, the CFRP hole wall experiences radial compressive stress and circumferential tensile stress, leading to shear plastic deformation or microcracking in the matrix under compression. Figure 12b displays the FNM-based crack propagation prediction paths for the top four plies, where the crack paths after element partition exhibit directionality consistent with the ply orientation. When the compressive stress from rivet expansion exceeds the critical value for fiber failure, localized fiber buckling and fracture occur around the hole. Additionally, delamination is distributed circumferentially around the rivet hole, with the most severe damage occurring at the Interply-1 interface. The delaminated area gradually decreases from the top ply to the bottom ply.

**Figure 13:**
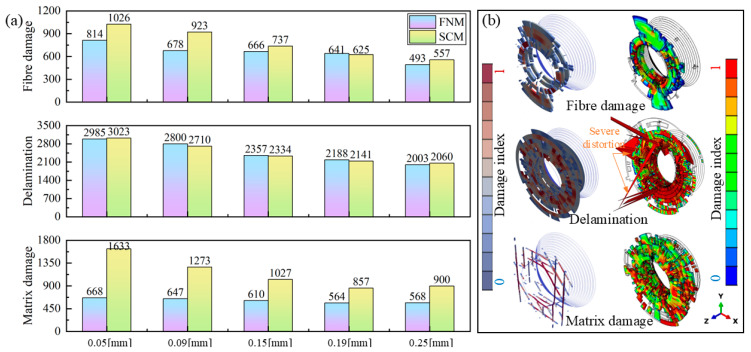
Comparison of simulated CFRP damage results between FNM and SCM: (**a**) number of damage elements at different clearance and damage modes, (**b**) comparison of riveting damage contours.

It is difficult to intuitively assess the effect of rivet-hole clearance on initial damage based solely on damage contour plots. Therefore, for quantitative analysis, the number of damaged elements satisfying failure criteria under different clearances was statistically evaluated, with FNM results compared against SCM results, as shown in Figure 13a. Here, damaged elements are defined as those with a damage index greater than 0.1 for the corresponding damage mode. Increasing the rivet-hole clearance reduces the number of damage elements. However, when clearance exceeds 0.19 mm, further enlargement provides limited mitigation of damage, and matrix damage elements even slightly increase. This suggests that a critical threshold is reached in the 0.19 mm to 0.25 mm range. Beyond this point, excessive clearance may result in insufficient material flow to fully fill the rivet-hole and form a proper joint, ultimately reducing the joint strength [8,16].

Figure 13b presents a comparison of riveting damage contours between FNM and SCM simulations. Morphologically, fiber and delamination damage patterns exhibit similarities in both models. However, the SCM results display element distortion due to deletion, particularly in cohesive elements representing delamination. Quantitatively, both methods yield comparable numbers of damaged fibers and delamination elements across different rivet-hole clearances, with notably minimal variation in delamination damage counts.

The most significant distinction lies in the representation of matrix damage. In the SCM, matrix damage manifests as cracks smeared in the form of damage indices in elements surrounding the hole, as these elements successively reach the cracking criterion. When the stress state of an element satisfies Equation (2), the constitutive relations at the integration points begin softening. The resulting crack propagates in a uniform and continuous manner and is highly dependent on the density and the orientation of the mesh [18]. Conversely, the FNM model directly simulates cracks as geometric discontinuities. By tracking crack tip progression and updating model topology, it distinctly visualizes discrete matrix crack paths and widths. This methodology provides more precise predictions of the progressive failure process governing matrix cracking during riveting.

### 3.4. Computational Efficiency

Computational efficiency is equally important as accuracy in simulation methodologies. Therefore, it is necessary to compare the computational performance of the SCM and DCM modeling approaches. The models of both methods are based on the ABAQUS/Explicit 2022 solver and were executed using a single core on the same machine (@ Intel(R) Xeon(R) CPU w5-3425 @ 3.19 GHz, 2023, Sapphire Rapids). After the solution, the physical time (Wallclock Time) elapsed from the start to the end of the model, and the processor time (CPU Time) consumed during computation, are used for evaluation.

**Figure 14:**
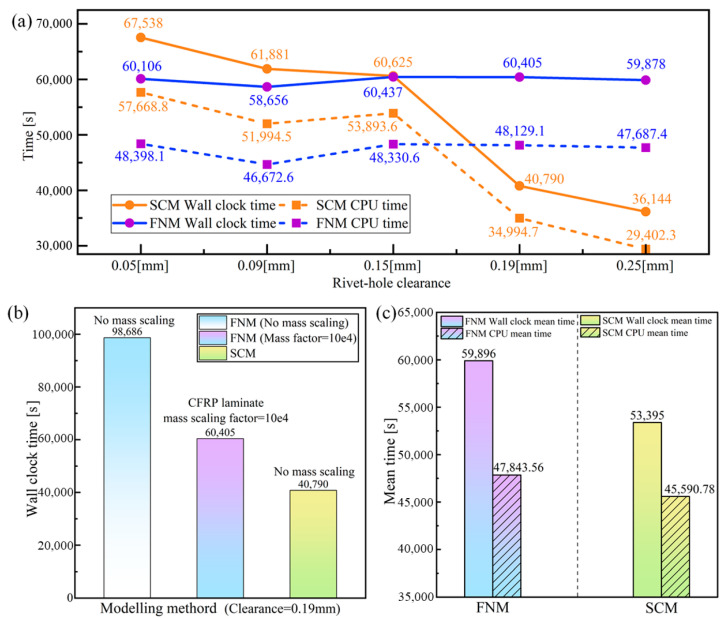
Comparison of computational efficiency between DCM and SCM: (**a**) Wallclock time comparison of DCM and SCM under different rivet-hole clearances, (**b**) effect of mass scaling on computational efficiency, (**c**) comparison of average Wallclock time between the DCM and SCM models.

Figure 14a summarizes the Wallclock Time and CPU Time results for both the FNM and SCM models under different rivet-hole clearances. Whether FNM or SCM, the variation trends of Wallclock Time and CPU Time with respect to the clearance are consistent. The computational cost of the SCM model decreases significantly with an increase in rivet hole clearance, while the FNM model computation time at different clearances does not change significantly. The difference between the two methods in computing time as the model changes can be attributed to the explicit integration algorithm’s stability being dependent on a dynamically adjusted critical time increment; the critical time increment Δt can be expressed as follows:

(22) $$\Delta t=\sqrt{A\frac{\rho }{E_{11}}}$$

where *A* is the in-plane area of the smallest element, *ρ* is the material density. In the explicit FE simulations of this study, the Courant condition was used as the convergence criterion [25], i.e., within a single time step, the distance over which physical information propagates cannot exceed the smallest element size.

For the SCM model, during the CFRP damage progression, localized softening induced by material failure may cause elements to experience excessive strain increments within a single time increment. The reduction in material modulus and wave velocity leads to a sharp decrease in the stable time increment. Additionally, element distortion and mesh degradation further increase the computational burden. Therefore, smaller rivet-hole clearances and larger rivet expansions cause more severe CFRP damage and element deformation (e.g., distorted cohesive elements shown in Figure 13b), extending the total simulation time. In contrast, for the FNM model, cracking onset is represented by a floating node partition element, without material softening. Although the reduced element size after partition (the mesh elements partitioning strategy shown in Figure 12) decreases the stable time increment, it also significantly reduces the risk of element distortion. As a result, the total computational time remains nearly unaffected by geometric model variations.

It should be noted that during the SCM-FNM comparison, mass scaling was applied to the CFRP laminate in the FNM model. Our previous studies [24] have shown that when the mass scaling factor is set to 10^4^, the FNM achieves an optimal balance between computational accuracy and efficiency. Figure 14b compares the computational time of the FNM model with mass scaling at the same rivet-hole clearance (c = 0.19 mm) against the FNM and SCM models without mass scaling. When the FNM model uses a mass scaling factor of 10^4^, its computational efficiency improves by 38.8% compared to the same FNM model without mass scaling. However, it still takes 1.48 times longer than the SCM model without mass scaling. A comparison of the average Wallclock time and CPU time required by both methods under different rivet-hole clearances (with the FNM model using a mass scaling factor of 10^4^) is shown in Figure 14c. In this case, the FNM model takes, on average, 1.12 times more computational time than the SCM model. When the clearance reaches 0.25 mm, the computational cost difference is at its maximum, with the FNM model requiring 1.66 times more time than the SCM model. Meanwhile, the CPU utilization rate (i.e., CPU time/Wallclock time) for FNM is 79.88%, and for SCM, it is 85.38%, indicating that the FNM model involves more I/O operations and idle waiting, resulting in lower overall CPU usage efficiency compared to the SCM model. These results confirm that although the FNM modeling provides a more intuitive and detailed simulation of crack initiation and propagation processes, it tends to reduce CPU utilization and increase the overall computational cost to some extent.

## 4. Conclusions

This study conducted both numerical simulations and experimental investigations on the damage behavior during the EMR process in aircraft CFRP structures. The differences in interference distribution, damage behavior, and computational efficiency between FNM and SCM riveting models under various rivet-hole clearances were analyzed. The main conclusions are as follows:

(1) The CFRP/Al double-sided countersunk riveting process can be divided into four stages. The axial interference distribution is non-uniform, with significantly higher interference sizes on the Al plate side than on the CFRP laminate side. Both the SCM and FNM models accurately predict the interference sizes on the laminate side but show deviations from experimental results on the Al plate side.
(2) The initial damage in CFRP/Al double-sided countersunk riveted joints primarily concentrates in the laminate’s straight hole part, while damage caused by rivet expansion rarely occurs in the countersunk hole part. Excessive normal stress in the fiber direction during riveting is the direct cause of fiber damage. The joint section fiber damage distribution predictions for FNM and SCM showed consistency; however, the degradation of the element stiffness in SCM resulted in severe element distortion around the hole.
(3) When the rivet-hole clearance increased from 0.05 mm to 0.25 mm, the number of fiber, matrix, and delamination damaged elements in the SCM model decreased by 45.71%, 31.86%, and 44.89%, respectively. And the corresponding numbers in the FNM model were 39.43%, 31.86%, and 14.97%. Increasing the clearance effectively reduces the damage severity, although this effect diminishes as the clearance increases beyond 0.22 mm. This suggests that there is an optimal clearance to minimize damage, although this is likely to depend on joint configurations and materials.
(4) Compared to the SCM model, the FNM model demonstrates enhanced accuracy in capturing the onset and propagation of matrix-dominated cracks. However, this improvement comes at the expense of computational efficiency. The FNM mode, with a mass scaling factor of 10^4^, requires 1.12 times more computation time than the SCM model. The FNM model’s CPU utilization is approximately 5% lower than that of the SCM model. Although FNM can track the crack propagation process with high fidelity, SCM has more advantages in engineering analyses of large-scale structures when efficiency is a priority.
(5) The specific selection of rivet-hole parameters still needs to be determined in future studies by considering both the static strength and fatigue performance of the joints. The results demonstrate that the FNM-based DCM approach can serve as an effective tool to support the manufacturing and design processes of CFRP structures. Due to the challenges in damage detection, this study does not address the issue of balancing prediction accuracy and computational efficiency. This issue will be further investigated in future work by comparing SCM and FNM in simulations based on material property-oriented benchmark problems.

## Figures and Tables

**Table 1:** Detail of rivet-hole clearance setups [unit: mm] [9].

	Group A	Group B	Group C	Group D	Group E
Nominal hole diameter *D*	4.08	4.12	4.18	4.22	4.28
Clearance *c*	0.05	0.09	0.15	0.19	0.25

**Table 2:** Contact pairs and friction coefficients settings [15,39].

Contact Property	Contact Surface Pairs	Friction Coefficient
Tangential behavior: Penalty Normal behavior: “Hard” Contact	Rivet die and rivet (driven head)	0.1
	Rivet and Al plate hole wall	0.1
	Lower Al plate and upper CFRP laminate	0.5
	Rivet and CFRP laminate hole wall	0.3

**Table 3:** ZT7H/QY9611 composite laminates’ mechanical properties [40,41].

Elastic Modulus			Shear Modulus		
*E*_11_	*E*_22_	*E*_33_	*G*_12_	*G*_13_	*G*_23_
146 GPa	10.4 GPa	10.4 GPa	6.12 GPa	6.12 GPa	3.45 GPa
Poisson’s ratio			Tensile strength		
*ν*_12_	*ν*_13_	*ν*_23_	*X_T_*	*Y_T_*	
0.278	0.3	0.42	2354 MPa	58 MPa	
Compressive strength			Shear strength		
*X_C_*	*Y_C_*		*S_L_*	*S_T_*	
1349 MPa	236 MPa		105 MPa	105 MPa	

**Table 4:** Delamination parameters in CZM [13,40,42,43].

Penalty Stiffness	Normal Interface Strength	Shear Interface Strength
*K*_n_	*N*	*S* = *T*
10^8^ GPa/m	60 MPa	95 MPa
Mode-I fracture toughness	Mode-II fracture toughness	Fracture criterion exponent
*G*_nc_	*G*_sc_	*η*
0.267 N/mm	0.75 N/mm	1.45

**Table 5:** Mechanical properties of 2A12 and TA1 [9].

Material	Mass Density (kg/m^3^)	Young’s Modulus (GPa)	Ultimate Tensile Strength (MPa)	Yield Strength (MPa)	Poisson’s Ratio
TA1	4510	108	353	260	0.34
2A12	7850	67.3	481	380	0.33

## Data Availability

The original contributions presented in this study are included in the article. Further inquiries can be directed to the corresponding authors.

## Notation and Abbreviations

*σ*_11_, *σ*_22_, *σ*_33_	normal stress components with local material coordinates
*τ*_12_, *τ*_23_, *τ*_31_	shear stress components with local material coordinates
*τ*_1_, *τ*_2_, *τ*_3_	interlaminar stress for the delamination
*N*, *S*, *T*	interlaminar interface strengths
*X*_T_/*X*_C_	longitudinal tensile/compressive strength
*Y*_T_/*Y*_C_	transverse tensile/compressive strength
*S*_T_/*S*_L_	transverse/longitudinal shear strength
*E*_11_/*E*_22_, *E_33_*	longitudinal/transverse elastic modulus
*G*_12_, *G*_13_, *G*_23_	shear modulus
*ν*_12_, *ν*_13_, *ν*_23_	Poisson ratio
*μ*_T_, *μ*_L_	friction-like parameters
*ε_fc_*, *ε^f^_fc_*	fiber longitudinal strain and final failure longitudinal strain
*ε_mc_*, *ε^f^_mc_*	matrix longitudinal strain and final failure longitudinal strain
*G_fc_*/*G_mc_*	fiber/matrix longitudinal fracture toughness
*σ_eff_*, *d_eff_*	effective stress and effective displacement
*d_f_*/*d_m_*	fiber/matrix damage index
*G_n_*, *G_s_*, *G_t_*	current fracture energies in the normal and shear directions
*G_nc_*, *G_sc_*, *G*_tc_	critical fracture energies in the normal and shear directions
*l*_e_	element length
*H*	fracture criterion exponent
*d*_n_	expanded rivet diameter
*T*_r_	reference temperature
${\dot{\varepsilon }}^{*}$	actual strain rate
CFRP	carbon fiber reinforced polymer
CT	computed tomography
SCM	smeared crack method
DCM	discrete crack method
DoF	degree of freedom
EMR	electromagnetic riveting
*K*_n_	penalty stiffness
*D*	rivet-hole diameter
*I*	interference size
*T*_m_	melting point temperature
$\dot{\varepsilon }$	static tensile strain rate
Al	aluminum alloy
SEM	scanning electron microscopy
CDM	continuum damage mechanics
FNM	floating node method
CZM	cohesive zone model
FEA	finite element analysis

## Appendix A

**Table A1:** Interference size measurement data.

		M1	M2	M3	M4	M5	M6	Average *d*_n_	Interference Size (*d*_n_ − D)/D × 100%
D = 4.08 mm (c = 0.05 mm)	Al	4.6950	4.5427	4.7903	4.6665	4.6855	4.6284	4.6681	14.41%
	CFRP	4.1998	4.2379	4.1236	4.1141	4.1808	4.3046	4.1935	2.78%
D = 4.12 mm (c = 0.09 mm)	Al	4.7903	4.6950	4.7426	4.6855	4.6474	4.5331	4.6823	13.65%
	CFRP	4.2379	4.1903	4.3522	4.5998	4.0379	3.9903	4.2347	2.79%
D = 4.18 mm (c = 0.15 mm)	Al	4.6950	4.6569	4.8664	4.7712	/	/	4.7474	13.57%
	CFRP	4.1331	4.2379	4.2189	4.3427	/	/	4.2332	1.27%
D = 4.22 mm (c = 0.19 mm)	Al	4.7860	4.7362	4.7807	4.7426	/	/	4.7614	12.84%
	CFRP	4.2671	4.2674	4.2951	4.2665	/	/	4.2740	1.28%
D = 4.28 mm (c = 0.25 mm)	Al	4.7183	4.767	4.8569	4.8284	4.8188	4.8474	4.8061	12.29%
	CFRP	4.2279	4.2003	4.3522	4.3046	4.3331	4.3712	4.2982	0.43%

During the preparation process, some specimens with clearances of 0.15 mm and 0.19 mm failed to be cut successfully, so only four data points are available (The M1–M6 unit is mm).
